# Editorial: Spores and Spore Formers

**DOI:** 10.3389/fmicb.2017.01046

**Published:** 2017-06-08

**Authors:** Imrich Barák

**Affiliations:** Department of Microbial Genetics, Institute of Molecular Biology, Slovak Academy of SciencesBratislava, Slovakia

**Keywords:** bacterial spores, *Bacillus subtilis*, Clostridia, initiation of sporulation, germination and dormancy, regulation of sporulation

Bacterial spore formers havebeen the focus of intense study for almost half a century. The most heavily studied of these is *Bacillus subtilis*, an internationally recognized model organism, whose physiology, biochemistry, and genetics have been studied for many years. Under nutrient rich conditions, *B. subtilis* grows and multiplies by a process of cell expansion followed by division at mid-cell to generate identical daughter cells; however, *B. subtilis* also has the ability to form spores, dormant cells which are resistant to many of the chemical and physical challenges that normally kill bacteria. Although many of the basic aspects of this process are now well-understood, bacterial sporulation still remains a highly attractive model for studying various cell processes at a molecular level. There are several reasons for this interest. First, some of the more complex sporulation steps are not fully understood or are only described using “controversial” models. Second, the extensive attention lavished on the unicellular development of *B. subtilis*, a single microorganism, has left us largely ignorant of the multitude of different sporulation mechanisms present in many other Gram-positive endospore and exospore formers; this diversity would likely increase if the sporulation processes of the Gram-negative spore formers were also included. The ability of spores to lie dormant and then germinate presents both threats and potential benefits to human health and welfare. Botulism and tetanus are infectious diseases transmitted by spores, while the spores of *Clostridium difficile* are responsible for hospital-acquired infections, which are harmful to patients and expensive to treat and eradicate. *Bacillus cereus* spores cause food poisoning and present a challenge to the food industry, while the spores of *B. anthracis*, the cause of anthrax, are a concern because of their potential use as bioterrorism and biowarfare agents. On the other hand, spore formers also have great potential in applied research. Spore forming bacteria are becoming increasingly important in the areas of probiotics, vaccine technology, and biotechnology. This Research Topic in *Frontiers in Microbiology* details the most recent advances in spore research basic science, covers emerging areas of scientific importance involving the use of spores, including their use as probiotics in humans and animals, and examines the use of spores as tools for nano-biotechnology where the spore surface can be used to efficiently display heterologous proteins. In addition, this Topic covers the ecological roles of spores, the taxonomy and systematics of spore forming bacteria, and the architecture and assembly of spores. This Research Topic was partially but not exclusively linked to the 7th European Spores Conference, held in Egham (UK) on 18–20 April 2016 (http://sporesconference.org/).

Generally, the sporulation process can be thought of as the last opportunity for the bacterial cell to endure when all other attempts to grow, compete, and survive have been exhausted. Sporulation initiation is a complex process in which different types of input, including nutritional, population density, and cell cycle signals, are integrated by the cell before the decision to sporulate is made. Sophisticated and sensitive signal transduction pathways are essential for accurately monitoring all these signals. In *B. subtilis* and most endospore-forming bacteria, sporulation initiation is under the control of a phosphorelay, an expanded two-component sensory signaling system, and the key molecule in transducing signals into the activation of those genes required for sporulation is the response regulator Spo0A. In this Research Topic, Lecca et al. analyse numerical simulations of a model of the gene network which regulates sporulation initiation in *Bacillus subtilis* to find that the non-linearity underlying the time series data is due to low-dimensional chaos. Pompeo et al. review the importance of serine/threonine protein kinases in sporulation regulation in *B. subtilis*.

The sporulation process is controlled at the gene expression level by a complex regulatory cascade composed of different compartment-specific sigma factors and additional regulator proteins, which are highly conserved among various spore formers. Despite this conservation, both regulatory and phenotypic differences are observed between different species of spore forming bacteria. Eijlander et al., demonstrate that deletion of the regulatory sporulation protein SpoVT results in a severe sporulation defect in *B. cereus*, but not in *B. subtilis*.

The resistance of spores to environmental attacks is attributable to the unique morphology of the spore, which is formed by several layers: the core, the cortex, the coat, the crust, and, in some cases, the exosporium. Dipicolinic acid (DPA), with its ability to bind Ca^2+^ ions, plays a key role in the dehydration and mineralization of the spore core. Dehydration of the spore core is the main factor underlying the resistance of spores to wet heat. The work of Jamroskovic et al. cataloged the heterogeneity in the heat resistance capacity and the overall DPA/calcium content in the spores of several *Clostridium* species. Edwards et al. characterized the chemical and stress resistances of *C. difficile* spores. Abhyankar et al. determined the influence of sporulation conditions on spore coat composition in *B. subtilis*. The spores of some *Bacillus* species are surrounded by the exosporium, an outermost surface layer. Lanzilli et al. clarified the *B. megaterium* exosporium permeability.

Under favorable conditions, spores germinate to produce vegetative cells that multiply. Brunt et al. characterized the diversity of germination apparatuses in different *C. botulinum* groups. Nagler et al. identified genes which are differentially expressed during *B. subtilis* germination in high-salinity environments. Besides sporulation, *B. cereus* can also undergo a differentiation process in which short swimmer cells become elongated and hyperflagellated swarmer cells which favor the migration of the bacterial community on a surface. Mazzantini et al. showed that the flagellar FlhF protein is required for the swarming motility and full pathogenicity of this bacterium.

## Author contributions

The author confirms being the sole contributor of this work and approved it for publication.

### Conflict of interest statement

The author declares that the research was conducted in the absence of any commercial or financial relationships that could be construed as a potential conflict of interest.

